# Electric Insulating Irrigations Mitigates Esophageal Injury Caused by Button Battery Ingestion

**DOI:** 10.3389/fped.2022.804669

**Published:** 2022-05-12

**Authors:** Wenyuan Jia, Guanghui Xu, Jiangang Xie, Luming Zhen, Mengsha Chen, Chuangye He, Xulong Yuan, Chaoping Yu, Ying Fang, Jun Tie, Haidong Wei

**Affiliations:** ^1^Digestive Diseases of Xijing Hospital, Fourth Military Medical University, Xi’an, China; ^2^Department of Emergency, Xijing Hospital, Fourth Military Medical University, Xi’an, China; ^3^Department of Anesthesiology, The Second Affiliated Hospital of Xi’an Jiaotong University, Xi’an Jiaotong University, Xi’an, China; ^4^Department of Gastroenterology, Xi’an Children’s Hospital, Xi’an, China

**Keywords:** button battery, foreign body, esophageal injury, edible oil, insulation

## Abstract

**Objective:**

Accidental ingestion of button batteries (BB), usually occurred in children and infants, will rapidly erode the esophagus and result in severe complications, even death. It has been recommended that treatment of this emergent accident as soon as possible with drinking of pH-neutralizing viscous solutions such as honey and sucralfate before surgical removal can mitigate the esophageal injury. Recently, we reported that the electric insulating solutions such as edible oils could mitigate tissue damage in BB-exposed esophageal segments. In this study, we compared the protective effect of kitchen oil with honey or sucralfate, the recommended pH-neutralizing beverages, and with their mixture on esophageal injury caused by BB ingestion in pig esophageal segments and in living piglets.

**Methods:**

Effect of olive oil irrigations was compared to that of honey or sucralfate irrigations in the BB-damaged esophageal segments freshly collected from the local abattoir and in live Bama miniature piglets with the proximal esophagus exposed to BB for 60 min. Also, the effect of olive oil and honey mixture (MOH) irrigations was assessed in live animals. The BB voltage was recorded before insertion and after its removal. Gross and histological analysis of the esophageal injury was performed after BB exposure in segmented fresh esophagus and 7 days after BB exposure in live animals, respectively.

**Results:**

Olive oil irrigations demonstrated better protective effect against BB-induced esophageal damage, compared to honey or sucralfate for BB-induced esophageal damage *in vitro*. But *in vivo* study showed that olive oil alone exacerbated esophageal injury because all esophagi irrigated with olive oil perforated. Surprisingly, irrigations with the MOH showed considerable protective effect for BB-induced esophageal damage in live animals, significantly better than irrigations with honey alone. The MOH decreased BB discharge, reduced area of surface injury, attenuated injured depth of esophageal wall thickness, and downed the mucosal injury index in comparison to using honey alone.

**Conclusion:**

Irrigations with olive oil alone couldn’t prevent the BB discharge and is harmful for BB ingestion before surgical removal. However, mixed with honey, olive oil very effectively prevents the BB discharging and produces better esophageal protection than honey.

## Introduction

With the frequent use of lithium button batteries in daily life, the incidence of button battery (BB) ingestions in children increases significantly (1, 2). Button battery (BB) ingestions would put children at great risks as it can cause serious esophageal damage, major complications, and high mortality (3–5). Removal of ingested BB should be performed as soon as possible, because esophageal injury can happen within 15 min after BB ingestion (6, 7) and complications increase rapidly with the delay of surgical removal (3, 8, 9). Administration of honey or sucralfate for the neutralization of the alkaline tissue environment caused by BB discharging before arriving at the hospital has been recommended to decrease the risk of tissue damage (7, 10, 11). In light of the original cause of esophageal damage, the generation of hydroxide at the negative pole from BB discharging (6), we recently tested the insulating strategy by edible oil irrigations *in vitro* to relieve the BB damage and found that edible oil was superior to weakly acidic juice irrigations in BB exposed esophageal segments (12). However, the effect of insulating strategy by edible oil was not compared with the recommended pH-neutralizing strategy using honey or sucralfate, and especially, the effect of edible oil on esophageal injury in live animals with BB ingestion was not verified yet. The present study was designed to answer these questions.

## Materials and Methods

All experimental protocols were reviewed and approved by the animal ethics committee of Xijing Hospital.

### *In vitro* Esophageal Injury

*In vitro* BB-induced esophageal injury was established as previously reported with little modifications (12). The fresh cadaveric porcine esophagus was collected from domestic pigs (Landrace, aged 3–4 months, weighing 24–27 kg) within 5 min after euthanization at the Abattoir of Wei River Bridge (Shannxi, China). The proximal 15 cm of each esophagus from 12 pigs was cut into 3 segments, 5 cm long for each. The esophageal segments were positioned on their length perpendicular to the ground with a plastic clip gently suspending the upper tip, and a button battery (Panasonic, CR2032, 20 mm in diameter, 3.2 mm in height, 3.0 V) was inserted into the middle of the esophageal segment cavity. Then the esophageal segments were repetitively irrigated with 10 ml of olive oil, honey or sucralfate, respectively. Each treatment group contained 3 esophageal segments. The esophageal segments were firstly repetitively irrigated in a 10 min time interval, then the irrigations with 30 min time interval was verified. Each solution irrigations were done by a 10-ml syringe without the needle from the top of the esophagus segments and the total volume was 110 ml with the 10 min interval and 90 ml with the 30 min interval.

The voltage of BB was measured using a voltmeter (Props Kitt MT-1509-Cn, China) before insertion and after removal. The tissue pH value was tested with an indicator paper (SIQI)^1^ before BB exposure and every 20 min after insertion for 2 h.

### *In vivo* Esophageal Injury

The *in vivo* BB-induced esophageal injury was established as previously reported (10). Bama miniature piglets weighing 9–12 kg (2 months old) were anesthetized with isoflurane (1–3%) and propofol (4–8 mg/kg for induction, 10 mg/kg/h for maintenance). The airway of animals was secured with endotracheal intubation. The piglets were placed in a supine position with its head backed at approximately a 30-degree angle. The hypopharynx was exposed with a Miller blade laryngoscope. Then the BB was placed in the proximal esophagus with its anode facing the posterior wall, and 10 ml saline was administrated in the esophagus. The BB remained in position for 60 min before it was retrieved. The BB voltage was recorded before placement and after removal, respectively.

Starting at the time 5 min after the BB placement, the esophagus was serial irrigated with either honey, olive oil or a mixture of olive oil and honey (MOH, with a volume ratio at 1:1) at a 10-min interval, respectively, until removal of the BB. Saline was used in control group. A total of 12 piglets were randomly allocated to the 4 treatment groups and each group contained 3 piglets. The irrigation solutions were delivered through a 4.5-mm endotracheal tube locked to the syringe. The irrigations were visualized endoscopically. Butorphanol was used (0.3 mg/kg subcutaneous once) before extubation for analgesia. The piglets were fed with normal diet after anesthesia recovery and supplemented with wet dog food, vegetables, and fruits.

### Esophagus Dissection for Histological Evaluation

On the seventh day, the piglets were euthanized using overdose phenobarbital (100 mg/kg, intravenously). The esophagus was exposed transthoracically and its proximal part was removed. After identification and photographing for the esophageal injury, the esophagus was fixed in 10% formalin.

### Histological Assessment

After fixation, all esophagi were trimmed and the injured parts together with distal internal normal controls were embedded with paraffin and sectioned to 4-μm thickness. Four transverse slices were chosen according to the quartile points along the injured length from each sample and subjected to hematoxylin and eosin staining. Histopathological changes were microphotographed under a light microscope. Index of esophageal injury was scored as described previously (12): 0, no obvious lesions; 1, lesions and inflammatory cell infiltration were observed in the mucosal layer; 2, lesions reached the submucosa layer, there was obvious patchy erosion, capillary dilation, as well as mucosal and submucosal neutral granulocyte infiltration; 3, lesions involved the muscular layer and a typical ulcer occurred. Depths of necrosis and granulation tissue as well as muscular injury length were measured using an ocular micrometer.

### Statistical Analysis

The statistical analysis was performed using SPSS 16.0 (SPSS Inc., Chicago, IL, United States) and GraphPad Prism 7 software (GraphPad Software, San Diego, CA, United States). Difference among groups were detected by one-way analysis of variance followed by the Newman–Keul’s test (multiple comparisons). Data were presented as the mean ± standard deviation. A *P*-value < 0.05 indicated a statistical difference.

## Results

### Olive Oil Irrigations Was More Effective Than Honey or Sucralfate *in vitro* in Prevention of Button Batteries-Induced Esophageal Injury

**FIGURE 1:**
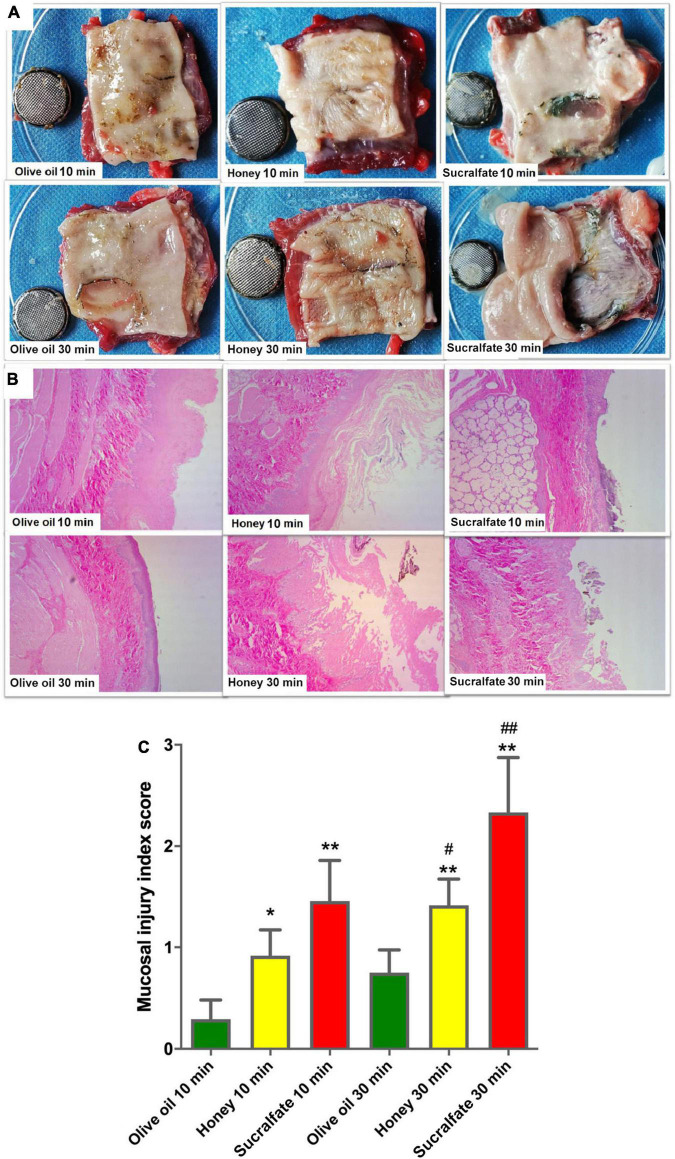
The effect of different treatments on esophageal injury after button-battery ingestion *in vitro* (*n* = 6). **(A)** The gross damage of segmented esophagus irrigated at 10 min (upper) or 30 min (lower) interval. **(B)** Hematoxylin and eosin staining of button battery-exposed esophageal segments after different treatments. **(C)** Mucosal injury index score based on hematoxylin and eosin staining. *P < 0.05 vs. Olive oil 10 min; ^#^*P* < 0.05 vs. Olive oil 30 min; **P < 0.01 vs. Olive oil 10 min; ^#^*P* < 0.01 vs. Olive oil 30 min.

The protective effect of olive oil irrigations was compared to honey or sucralfate in BB-exposed porcine esophageal segments. The serial irrigations were done with two different time intervals: 10 and 30 min after the BBs were put in the cavity. The gross damage was shown that esophagi with olive oil irrigations had the minimal injury as compared to honey or sucralfate (Figure 1A). Histological examination in hematoxylin and eosin-stained sections was shown in Figure 1B. The mucosal layer of olive oil-irrigated esophageal segments with 10 min interval for irrigating was intact, and that was almost intact with 30 min interval, while the mucosal layer in honey or sucralfate-irrigated group was in part (with 10 min time interval) or completely destroyed (with 30 min time interval). The mucosal injury index scores of treated esophagi were presented in Figure 1C. In groups of 10 min interval irrigations, olive oil administration had the lowest mucosal injury index score than that irrigated by honey or sucralfate (*P* < 0.05). Meanwhile, even irrigated in 30 min interval, olive oil was also more effective than the honey or sucralfate groups (*P* < 0.05).

### Olive Oil Prevented Tissue Alkali Production From the Button Battery Discharging in Segmented Porcine Esophagi

**FIGURE 2:**
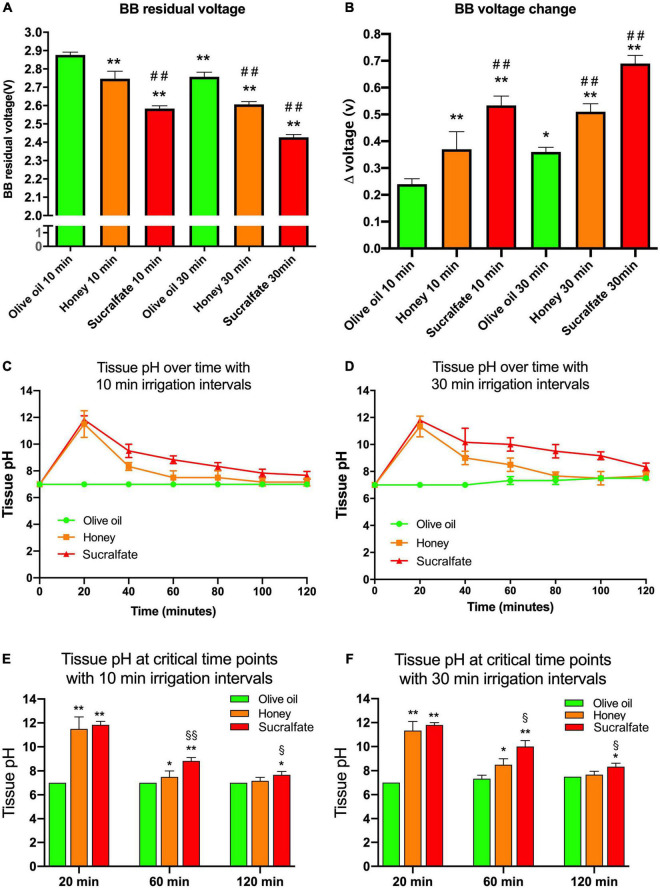
The button battery discharge and esophageal tissue alkalization after different treatments *in vitro* (*n* = 6). **(A)** The residual voltage of button batteries after esophageal injury. **(B)** The discharged voltage of button batteries after esophageal injury. **(C)** The change of the tissue pH value of esophagi treated with 10 min time interval irrigations. **(D)** The change of the tissue pH value of esophagi treated with 30 min time interval irrigations. **(E)** The tissue pH value at critical time points of esophagi treated with 10 min time interval irrigations. **(F)** The tissue pH value at critical time points of esophagi treated with 30 min time interval irrigations. *P < 0.05 vs. Olive oil 10 min group; **P < 0.01 vs. Olive oil 10 min; ^##^*P* < 0.01 vs. Honey 10 min; ^§^*P* < 0.05 vs. Honey 30 min; ^§§^*P* < 0.01 vs. Honey 30 min.

The BB discharge and injured esophageal tissue pH value were measured, as shown in Figure 2. Olive oil administration repeated every 10 min increased the residual voltage of BB compared to either honey or sucralfate (Figure 2A, *P* < 0.01). Meanwhile, when irrigated with 30 min intervals olive oil also increased the residual voltage of BB compared to either honey or sucralfate (Figure 2A, *P* < 0.01). The voltage change of inserted BB was presented in Figure 2B. Olive oil irrigations with either 10 or 30 min intervals could both decrease the voltage discharge of BB as compared to honey or sucralfate (Both *P* < 0.01). The pH value of damaged tissue surface over time after BB exposure was shown in Figures 2C,D. Irrigations with olive oil prevented the increase of tissue pH value shortly after BB exposure. The tissue pH at critical time points were shown in Figures 2E,F. Olive oil irrigations prevented the increase of tissue pH at 20 min after BB exposure as compared to honey or sucralfate with either 10 or 30 min intervals, and then maintained a neutral environment.

### *In vivo*, Irrigating the Esophagus With Olive Oil Alone Exacerbated Button Batteries-Induced Damage; While Mixing Olive Oil With Honey, Irrigations Effectively Protected the Esophagus From Button Batteries-Induced Damage by Preventing the Button Batteries Discharging

**FIGURE 3:**
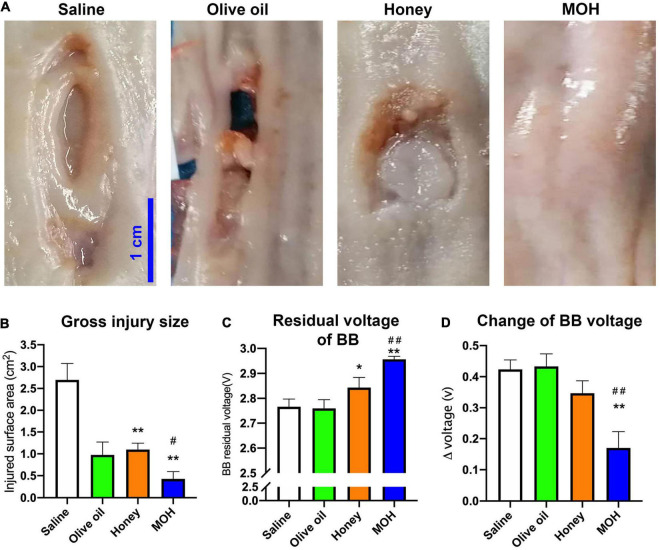
The *in vivo* effect of different treatments on button battery exposed esophagi (*n* = 3). **(A)** The gross injury of damaged esophagi immediately removed from live animals 7 days after the battery exposure. **(B)** The surface injury size of damaged esophagi. **(C)** The residual voltage of button batteries after 60 min exposure. **(D)** The discharges of placed batteries. *P < 0.05 vs. Saline; ^#^*P* < 0.05 vs. Honey; **P < 0.01 vs. Saline; ^##^*P* < 0.01 vs. Honey.

Irrigating with olive alone or MOH for the protection of BB-induced damage before removal was compared to honey alone irrigations, as shown in Figure 3. The esophageal injury was photographed with a ruler immediately after removal from piglets (Figure 3A). Saline was served as the control solution. Saline treatment resulted in a deep and large tissue damage. All animals treated with olive oil alone had an esophageal perforation. Irrigating with honey alone reduced the damage of the esophagus in comparison to the saline group. When irrigating with MOH, the esophageal damage was further mitigated. The gross injury surface size was shown in Figure 3B. Honey irrigations reduced the injury size compared to saline (*P* < 0.01). However, piglets irrigated with MOH had a milder esophageal injury area compared to honey alone (*P* < 0.05). The BB voltage was measured as shown in Figure 3D. Compared to saline, both honey (*P* < 0.05) and MOH (*P* < 0.01) increased the residual voltage of the BB. Regarding to the BB voltage, only MOH significantly prevented the discharge of BB as compared to saline (*P* < 0.01) or honey alone (*P* < 0.01). Unfortunately, olive oil irrigations had no effect in preventing BB discharge compared with saline.

### Use of Edible Oil Is Ineffective Even Harmful, While Mixture of Olive Oil and Honey Irrigations Improved the Esophageal Histological Manifestations, Even Better That Use of Honey Alone in Button Batteries-Ingested Piglets

**FIGURE 4:**
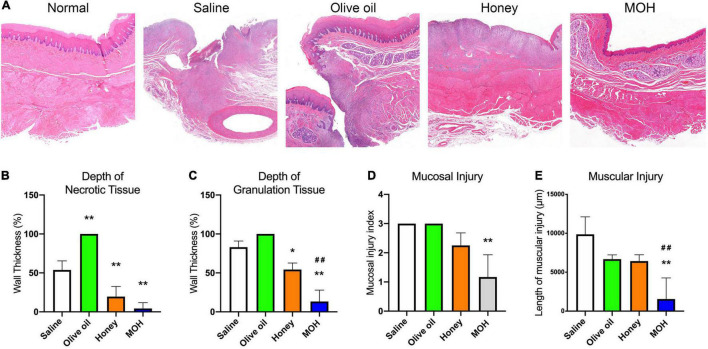
The histological assessment of the damaged esophagi *in vivo* by the button battery ingestion (*n* = 3). **(A)** Hematoxylin and eosin staining of the battery-damaged esophagi. **(B)** The necrosis depth of esophageal wall. **(C)** The depth of granulocytes infiltration into the esophageal wall. **(D)** The mucosal injury index based on hematoxylin and eosin staining. **(E)** The muscular injury length of the damaged esophageal tissue. *P < 0.05 vs. Saline; **P < 0.01 vs. Saline; ^##^*P* < 0.01 vs. Honey.

The representative hematoxylin and eosin staining of damaged esophagus from live animals in each group was shown in Figure 4A. In saline treated esophagus, there was deep necrosis and almost all layers of esophageal wall were severely damaged. Unexpectedly, in olive oil treated esophagus, the critical perforating injury was seen. Honey-treated esophageal showed the disappearance of mucosa layer and moderate granulocytes infiltration. However, in MOH treated esophageal, there was an almost intact mucosa layer and only mild granulocytes infiltration. The depth of necrosis was shown in Figure 4B. Compared to saline, olive oil alone lead to a transmural damage (*P* < 0.01), while honey (*P* < 0.01), or MOH (*P* < 0.01) limited the necrosis of esophageal wall. The depth of granulation was also analyzed as shown in Figure 4C. There was a transmural granulation in olive oil alone treated esophagus. But honey (*P* < 0.05) or MOH (*P* < 0.01) treatment mitigated the granulation of the esophageal wall as compared to saline. Besides, animals irrigated with MOH had minimized esophageal granulation compared to honey alone (*P* < 0.01). In addition, the mucosal injury index and the length of muscular injury were also analyzed (Figures 4D,E). Only the MOH treated esophagus had a lower injury index compared to saline control (*P* < 0.01). As to muscular injury, only MOH irrigations reduced the injury length as compared to saline (*P* < 0.01). Meanwhile, the muscular injury length in MOH-treated esophagus was shorter than honey alone treated esophagus (*P* < 0.01).

## Discussion

In the present study, we first compared olive oil irrigations with the currently recommended pH-neutralizing strategy with honey or sucralfate in the BB ingestion model *in vitro*, and we showed that olive oil irrigation was the most effective method for the protection of esophageal injury *in vitro*. However, when tested *in vivo*, it turned out that esophageal irrigations with olive oil alone in case of BB ingestion before removal was very dangerous and should be completely avoided. Nevertheless, we surprisingly found that insulating strategy with the olive oil-containing viscous solution MOH for the mitigation of BB-induced esophageal damage before surgical removal was considerably effective.

Particular efforts should be taken for BB ingestion in children or infants as the incidence is increasing; the major complications and mortality remaining high (13). Ingestion of BB in children or infants is a real emergency situation (14). Once ingested, the BB will discharge on the contacting surface of esophageal mucosa, leading to production of alkali on the negative pole and causing tissue necrosis very quickly (6, 15). Delayed treatment will result in more discharge of the BB, accumulation of larger amount of alkali, and much severer and terrible damage of the esophagus. It is recommended that once diagnosed, the BB should be removed as soon as possible (9, 11). Besides, the treatment before surgical removal is also of great importance as the pH-neutralizing strategy can mitigate the esophageal injury (10). The work by Anfang et al. demonstrated that using honey or sucralfate to neutralize the high pH in the contacted tissue shows a promising effect (10). However, we argue that rather than neutralizing the already produced alkali that causes tissue necrosis, we might be able to prevent the discharging of BB with insulating liquids from the beginning. Thus, we previously tested the edible oils *in vitro* with segmented porcine esophagi in light of insulating strategy, and found that edible oils did prevent the discharge of ingested BB and were very effective in prevention of the esophageal injury caused by BB exposure (12). We then compared the insulating strategy with pH-neutralizing strategy *in vitro* and *in vivo* in the present study. Insulating strategy using olive oil showed a much considerable effect *in vitro* as compared to honey or sucralfate, with the most minimal esophageal damage, as well as the least BB discharge. However, the impact of saliva, gastroesophageal reflux, and the esophageal peristalsis on the tissue damage after BB ingestion could not be evaluated in cadaveric esophagus, as ions and digestive enzymes in saliva, refluxed gastric acid and the esophageal movement could all aggravate the damage. Therefore, it was very essential to carry out the *in vivo* experiment. However, when transferred to *in vivo*, esophageal irrigations with olive oil alone resulted in perforating damage and failed to prevent the BB discharge, even worse than saline irrigations, suggesting that olive oil alone irrigating is very dangerous in BB ingested children or infants before removal. Nevertheless, we still wonder why all animals irrigated with olive oil had an esophageal perforation. We think that the failure of olive oil irrigations is due to its low viscosity that after irrigated into the esophagus the olive oil couldn’t remain around the BB for long but flew away because of esophageal peristalsis (16). As the residual olive oil partially coated the BB, the injured esophageal surface area was decreased. However, the BB could still discharge. Since the contacted area was decreased the BB would discharge on a smaller area. Then the discharged power on the smaller area would cause deeper tissue injury compared to a larger contacted area. In light of such inference, we considered to increase the viscosity of olive oil simply by mixing it with honey as honey is almost 50 times viscous as olive oil at body temperature.^2^ The significant effect of the olive oil and honey mixture verified our idea. The mixture not only most effectively prevented the discharging of BB *in vivo*, but also markedly mitigated the esophageal injury, even much more effectively than honey. The results strongly suggest that viscous insulating liquid irrigations for the treatment of BB ingestion before surgical removal could be useful.

We used the porcine model according to the previously reported BB-induced esophageal injury *in vivo* (10). The model of esophageal injury in pigs can well mimic human esophageal injury, because they not only share common esophageal anatomy including similar size and thickness of the esophageal layers from squamous epithelium to the outer muscle layers and adventitias including the esophageal submucosal glands in the proximal part (17), but also they have the similar the histological changes after endoscopic submucosal dissection (ESD) with the presence of a layer of myofibroblast in the floor of ulcers (18). However, there are some differences in composition of esophagus between porcine and humans, the muscularis propria is mainly skeletal muscle in pigs while is smooth muscle in humans, and pigs have a keratinized layer above the squamous epithelium which is uncommon in human. And the longitude directions of esophagus are different to the direction of gravity. These differences might affect the severity of esophageal injury caused by BB, but porcine esophagus still represents a superior animal model for human beings (19).

Although we surprisingly found out the significant protection of the mixture for BB-induced esophageal injury, a lot of questions should be addressed like the ratio of two component, the tastes, irrigation volume, irrigation frequency and complicating of anesthesia management (20). In the study, we used 1:1 ratio for the mixing. The two liquids can be mixed evenly by using household tools such as agitators or whisks, and the mixed MOH was irrigated immediately after preparation. However, the mixed MOH started to separate a few minutes later. Nevertheless, the two liquids were not completely separated, and separation process took about 30 min to 1 h, much longer than the time interval for serial irrigations. The conductivity of honey would be reduced by the successfully mixed oil, but the proportion of the two liquids should be optimized. As for the taste, olive oil is less pleasant than honey for children. However, as for the smell, olive oil is of some aroma, and its intake has many health benefits for children (21). Adding olive oil to honey can help increase its intake, while oral administration of honey is also recommended for other medical needs, such as postoperative analgesia for tonsillectomy and oral mucositis treatment in children (22, 23). Honey contains 65–80% (w/w) of glucose and fructose (24), and it still tastes very sweet even diluted twice, sweeter than the aqueous solution with same content (w/w) of sucrose, because sucrose (a disaccharide) is with lower sweetness than monosaccharide glucose and fructose. Olive oil can also increase the spread of honey in the mouth after oral intake and have a special aromatic smell. In fact, MOH tastes almost as sweet as honey. Therefore, oral MOH is very likely to be accepted by children. Whether a different volume will protect the esophagus better or a smaller volume provide same protection is undetermined, as increasing volume might increase the aspiration risk as well as esophageal peristalsis (25). As for the frequency, we prolonged the time interval to 20 min, it seemed that the prolonged irrigation interval was no better than 10 min interval (data not shown). However, whether a slighter increase of the interval duration will produce comparable or even better protection of the esophagus still need further verification. Nevertheless, considering its most effective protection and minimized damage, the insulating strategy with the mixture should be tried as much as possible. Indeed, the ingestion of BB in children is a real emergency, and should be treated without delay. Therefore, it is recommended anesthesia should not be suspended because of the nil per oral guidelines (14), and the surgical removal must be performed as soon as possible after the confirmative diagnosis. A carefully prepared anesthesia with considerations for full stomach patients should minimize the pulmonary aspiration risk. Besides, the already damaged esophagus with conceivable severe complications should be very cautiously weighed against the aspiration risk (20, 26). However, in order to decrease the aspiration risk, the irrigation volume and time interval should be very carefully optimized in future.

## Data Availability Statement

The raw data supporting the conclusions of this article will be made available by the authors, without undue reservation.

## Ethics Statement

The animal study was reviewed and approved by the animal ethics committee of Xijing Hospital (Xi’an, China).

## Author Contributions

WJ, GX, and JX conceived of the study and drafted the manuscript. WJ, GX, JX, and LZ designed the experiments. WJ and GX performed the pathology experiments. LZ and CH performed the *in vivo* model. XY and CY performed the biophysical examinations. HW, YF, and JT analyzed the data, discussed and reviewed the manuscript. All authors agreed the final version of the manuscript.

## Conflict of Interest

The authors declare that the research was conducted in the absence of any commercial or financial relationships that could be construed as a potential conflict of interest.

## Publisher’s Note

All claims expressed in this article are solely those of the authors and do not necessarily represent those of their affiliated organizations, or those of the publisher, the editors and the reviewers. Any product that may be evaluated in this article, or claim that may be made by its manufacturer, is not guaranteed or endorsed by the publisher.

## Footnotes

1
https://item.jd.com/10711086311.html

2
https://wiki.anton-paar.com/en/flower-honey-blended/
